# Investigating the Relationships among Stressors, Stress Level, and Mental Symptoms for Infertile Patients: A Structural Equation Modeling Approach

**DOI:** 10.1371/journal.pone.0140581

**Published:** 2015-10-20

**Authors:** Jong-Yi Wang, Yi-Shan Li, Jen-De Chen, Wen-Miin Liang, Tung-Chuan Yang, Young-Chang Lee, Chia-Woei Wang

**Affiliations:** 1 Department of Health Services Administration, China Medical University, Taichung, Taiwan; 2 Department of Business, Chungyo Department Store, Taichung, Taiwan; 3 Office of the President, National Changhua University of Education, Changhua, Taiwan; 4 Graduate Institute of Biostatistics, China Medical University, Taichung, Taiwan; 5 Reproductive Medicine Center, Department of Obstetrics and Gynecology, China Medical University Hospital, Taichung, Taiwan; 6 Reproductive Medicine Center, Yuan's General Hospital, Kaohsiung, Taiwan; 7 Department of Obstetrics and Gynecology, Taipei Medical University Hospital, Taipei, Taiwan; Chiba University Center for Forensic Mental Health, JAPAN

## Abstract

**Objective:**

Patients with infertility are a high risk group in depression and anxiety. However, an existing theoretically and empirically validated model of stressors, stress, and mental symptoms specific for infertile patients is still a void. This study aimed to determine the related factors and their relational structures that affect the level of depressive and anxiety symptoms among infertile patients.

**Methods:**

A cross-sectional sample of 400 infertility outpatients seeking reproduction treatments in three teaching hospitals across Taiwan participated in the structured questionnaire survey in 2011. The hypothesized model comprising 10 latent variables was tested by Structural Equation Modeling using AMOS 17.

**Results:**

Goodness-of-fit indexes, including χ^2^/DF = 1.871, PGFI = 0.746, PNFI = 0.764, and others, confirmed the modified model fit the data well. Marital stressor, importance of children, guilt-and-blame, and social stressor showed a direct effect on perceived stress. Instead of being a factor of stress, social support was directly and positively related to self-esteem. Perceived stress and self-esteem were the two major mediators for the relationships between stressors and mental symptoms. Increase in social support and self-esteem led to decrease in mental symptoms among the infertile patients.

**Conclusions:**

The relational structures were identified and named as the Stressors Stress Symptoms Model, clinically applied to predict anxiety and depression from various stressors. Assessing sources and level of infertility-related stress and implementing culturally-sensitive counseling with an emphasis on positive personal value may assist in preventing the severity of depression and anxiety.

## Introduction

The trend toward fewer children has been very severe in most of the industrialized countries and also in many developing countries [1, 2]. The total fertility rate (TFR) of Taiwan in 2010 was 0.90, ranked the lowest in the world, and averaged 1.07 in the recent 5 years [1, 3]. The occurrence of infertility is believed to impact on TFR [4]. Nevertheless, numerous individuals with infertility are still seeking reproduction treatments, striving for the chance of conception. Therefore, healthcare organizations worldwide face an imperative shared challenge of caring and treating infertile patients, which carries the implications of health promotion.

Infertility is an escalating health issue, affecting more than 10% of couples globally [4, 5]. The experience of infertility is highly stressful for individuals in the duration of infertility [6]. Patients diagnosed with infertility and individuals involved would undergo substantial psychological stress, although varied, during the treatment process [7]. Psychological stressors implicated in the infertility experience involve negative self-image, sense of guilt and self-blame, and other psychosocial factors [8–11]. Consequently, mental symptoms might emerge among people under this stressful condition. Existing literature further identified depression and anxiety as two of the most frequent mental disorders among patients with infertility [7, 10, 12–14]. Compared with fertile counterparts, infertile patients showed higher or approximately double of the odds of psychological distress, making them a vulnerable population [7, 15]. More specifically, the overall prevalence rate of depression among infertile couples reported by a meta-analysis study was 47% [16]. Seeing that stress and even mental illness are a widespread and costly problems [17] especially for individuals with infertility, mental disease prevention and well-being advancement have become critical. From another perspective, since findings from previous research asserted that emotional conditions was a predictor of treatment outcome for in vitro fertilization/embryo transfer technology (IVF/ET) [18], a concern for psychological well-beings hence delivers clinical implications in infertility medicine.

Study of stress may comprise stressors (source of stress) [19] and stress reactions [20]. In addition to the aforementioned possible stressors, research revealed that infertility-related factors including perceived importance of children, social stressor, and marital stressor might expose infertile individuals to a high stress level [6, 21, 22]. The stressors described so far could be conceptually categorized into personal (i.e. importance of children, guilt-and-blame, etc.), marital, and social ones, concluded from prior research [21]. Study further indicated that self-concept acted as a mediator in the relationship between social support and depressive symptoms [23]. Hence, it can be presumed that self-esteem is a mediator between social support and mental symptoms. The two relational paths were integrated into the following conceptual model accordingly. Regarding stress reactions, previous study suggested that depression and anxiety might display an asymmetric relationship [24], the unidirectional effect from depression to anxiety. Because the predictors of depression and anxiety among infertile patients remains obscure [25], systematically constructing a useful explanatory model is very demanding in this field. An existing theoretically and empirically validated model of stressors, stress, and mental symptoms specific for infertile patients is still a void.

**Fig 1:**
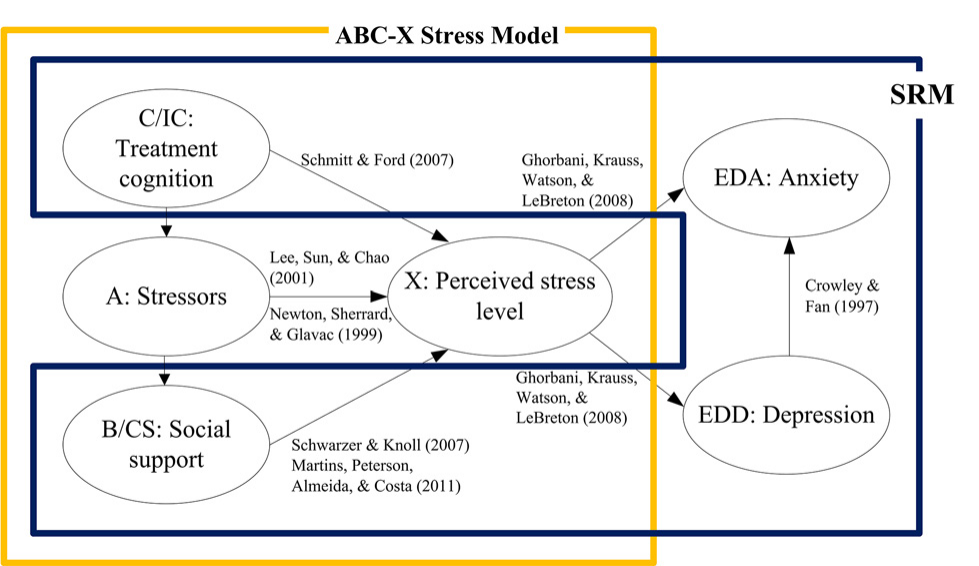
Theoretical Framework: Factors of Stress, Stress Level, and Mental Symptoms among Patients with Infertility.

Two existing theories including the ABC-X Stress Model [26] and the Self-Regulation Model (SRM) [27, 28] mainly contributed to the theoretical skeleton of the present research. The ABC-X Stress Model consists of stress event (A), family resources (B), cognition (C), and degree of stress (X). This model asserted that the degree of stress (X) depends on the effect of family resources (B) and cognition (C) on stress event (A). Adapting the ABC-X stress model for the depiction of the perceived stress level, the current study proposed the diagnosis of infertility being the stress event (A), social support served as B, and treatment cognition as C [29]. SRM, however, addressed three latent variables pertaining to psychological processes in infertility, including cognitive representation of infertility (IC), use of coping strategies (CS), and emotional distress and well-being (ED) that could be applied to further extend the outcome observation to mental illness. Cognitive representation includes consequences and controllability of infertility treatments; use of coping strategies contains problem management; emotional distress and well-being as the major outcome can be represented by mental symptoms including depression and anxiety [28]. The ABC-X Stress Model and SRM Model were integrated through the overlapped components including treatment cognition (C/IC) and social support (B/CS), coupled with additional variables and relationship links supported by empirical studies as illustrated in Fig 1. This study proposed a theoretical framework that explains the relationships among the synoptic factors of stress, perceived stress level, and mental symptoms specific to patients with infertility. Perceived stress is viewed as a mediator for the effects of treatment cognition, stressors, and social support on depression and anxiety (Fig 1). Since the aforementioned two models did not aim to describe stressors, the present study incorporated the infertility-related stressors into the theoretical framework.

In view of the high risks of depression and anxiety for infertile patients and also lack of research exploring the relationships among stressors, stress level, and mental symptoms, this study sought to examine and establish the structural path model by using Structural Equation Modeling (SEM), which highlights strong evidences for causal relationship among latent variables.

## Methods

### Hypothesis and research design

**Fig 2:**
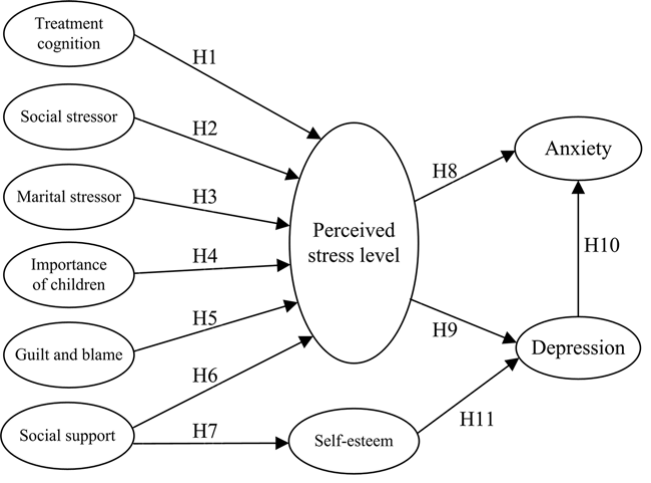
Initial Hypothetical Model.

It was hypothesized that the infertility-related stressors may induce depression or anxiety through the mediating effect of perceived stress level and self-esteem among patients with infertility. Theoretical model constructed in this study is presented in Fig 2 that depicts detailed stressors described previously and totally 11 individual hypothetical relationship paths (H1 to H11) to be tested. Derived but different from the ABC-X Stress Model and SRM Model, the hypothetical model substantially highlights the factors of stress. The hypothesis was tested in a cross-sectional design, analyzing the survey data collected from various locations in Taiwan.

### Participants

400 outpatients were selected using a mixed sampling method from infertility clinics of reproductive medicine centers at three teaching hospitals, including one medical center and two regional hospitals in northern, central, and southern Taiwan. First, by using a simple random sampling method, this study selected one session out of a clinic time frame based on daytime and nighttime and Monday to Friday schedule to fully represent outpatient characteristics. Subsequently, infertile outpatients in the selected session were invited to this multi-site study. The same time-sampling method was repeated and applied to all the three reproductive medicine centers. Sample size was first determined by calculating the equations according to the prevalence of mental illness among infertility patients on the basis of previous research [7, 12, 30]. The sample size was proved to be sufficient, reaching the satisfactory 80% power. The proposed research was approved by the Institutional Review Board at China Medical University Hospital, Institutional Review Board of Taipei Medical University Hospital, and Institutional Review Board of Yuan's General Hospital. Informed consent forms were signed by all voluntary respondents. Participation in this survey was completely anonymous. All respondents were solicited for completing the questionnaires when awaiting reproductive health care. Respondents were notified the provision of small material rewards upon completion of the questionnaires. Consequently, a total of 400 patients responded to the questionnaires and all were valid samples. All data were collected in 2011.

Table 1 presents the descriptive characteristics of the study sample. Overall, the majority of the outpatients with infertility in this study were female (94.0%, man accounted for only 6%: 24/400), in middle social economic status (47.5%), married within recent 1–5 years (65.5%), without any child (over 90%), and diagnosed infertility within recent 1–3 years (60.0%). Slightly lower than half of the participants were aged 31–35 years (44.3%). Regarding health and medical characteristics of the patients studied, self-perceived health status showed that slightly more than half of the participants considered themselves healthy (51.0%); nearly one third reported diseases other than infertility (comorbidity at 36.1%); the majority experienced psychological discomforts (91.4%); slightly more than half of the patients were under medication (54.5%); nearly three out of four (74.1%) underwent either Artificial Insemination by Husband (AIH) or IVF/ET.

**Table 1:** Characteristics of the Samples Studied (N = 400).

Variables		N / Mean	% / SD
**Gender**			
	Female	376	94.0
	Male	24	6.0
**Age (year)**			
	25–30	42	10.5
	31–35	177	44.3
	36–40	126	31.5
	41–45	53	13.3
	46–50	2	0.5
**Social economic status (SES)**			
	Low	89	22.3
	Middle	190	47.5
	High	121	30.3
**Years of marriage**			
	Less than 1 year	6	1.5
	1–5 years	262	65.5
	6–10 years	117	29.3
	More than 6 years	15	3.8
**Number of male children**			
	0	386	96.5
	1	14	3.5
**Number of female children**			
	0	377	94.3
	1	19	4.8
	2	4	1.0
**Self-perceived health status**			
	Very healthy	37	9.3
	Healthy	204	51.0
	Moderate	135	33.8
	Unhealthy	23	5.8
	Very unhealthy	1	0.3
**Comorbidity**			
	Hypertension	10	2.4
	Heart disease	3	0.7
	Diabetes	8	1.9
	Chronic arthritis	6	1.4
	Liver disease	6	1.4
	Gastrointestinal disease	74	17.5
	Asthma	10	2.4
	Kidney disease	2	0.5
	Hyperlipemia	9	2.1
	Other	25	5.9
	None	271	63.9
**Self-reported psychological condition (Including fatigue, sleeping problems, anxiety, frustration, helplessness, anger, tension, etc)**			
	Present	324	91.4
	Absent	76	8.6
**Years of infertility**			
	Less than 1 year	28	7.0
	1–3 years	240	60.0
	4–6 years	83	20.8
	7–9 years	28	7.0
	10 years or more	21	5.3
**Current Medication**			
	Regular infertility prescription	168	42.0
	Regular non-infertility prescription	24	6.0
	Other	26	6.5
	None	182	45.5
**Current treatment**			
	Preliminary assessment	73	18.3
	Medication	27	6.8
	Artificial insemination by husband (AIH)	141	35.3
	in vitro fertilization/embryo transfer (IVF/ET)	155	38.8
	Other	4	1.0
**Psychological constructs in the structural model (Fig 2)**			
	Treatment cognition (averaged from 5 items) ^1^	3.707	0.635
	Social stressor (averaged from 3 items) ^2^	3.332	0.684
	Marital stressor (averaged from 4 items) ^3^	2.613	0.875
	Importance of children (averaged from 5 items) ^4^	3.543	0.757
	Self-esteem (averaged from 3 items) ^5^	4.044	0.606
	Guilt-and-blame (averaged from 6 items) ^6^	2.754	0.863
	Social support (averaged from 6 items) ^7^	1.780	0.578
	Stress level (averaged from 4 items) ^8^	2.064	0.647
	Anxiety (Summed from 7 items)	6.148	3.463
	Depression (Summed from 7 items)	4.525	3.393

^1^ Cronbach’s α = 0.85; factor loading > 0.57.

^2^ Cronbach’s α = 0.63; factor loading > 0.64.

^3^ Cronbach’s α = 0.83; factor loading > 0.65.

^4^ Cronbach’s α = 0.84; factor loading > 0.63.

^5^ Cronbach’s α = 0.78; factor loading > 0.73.

^6^ Cronbach’s α = 0.87; factor loading > 0.55.

^7^ Cronbach’s α = 0.83; factor loading > 0.61.

^8^ Cronbach’s α = 0.83; factor loading > 0.69.

### Research instrument

This study utilized six existing well-established questionnaires to measure corresponding constructs; definition of constructs, number of items, and detailed description of scale are presented as follows:

1. Brief Illness Perception Questionnaire (BIPQ; Weinman, Petrie [31]): Treatment cognition
  BIPO was adapted to measure treatment cognition, which was defined as the understanding of infertility treatment options and procedures, probability of conception, and channels of treatment information for infertility. Five items of treatment cognition were used in the survey. Each of the items was scored from 1 (strongly disagree) to 5 (strongly agree).
2. Fertility Problem Inventory (FPI; Newton, Sherrard [21]): Social stressor, marital stressor, and importance of children
  Items of FPI were applied to elicit participants’ experiences of social stress, marital stress, and their beliefs in the importance of children. Social stressor refers to the source of social pressures stemming from relationships with other people and from social interaction: items concerning individual’s being asked of questions about children as well as the attitude of other family members toward children. Marital stressor reflects the source of stress provoked by lacking of understanding and support in martial relationship when facing the infertility problems. Importance of children pertains to personal concerns from parenthood for a life without child and thus in the fear of losing meaning of life due to childlessness. There were six items in each individual subscales which measured social stressor, marital stressor, and importance of children. All the items were scored from 1 (strongly disagree) to 5 (strongly agree).
3. Infertility Questionnaire (IFQ; Bernstein, Potts [32]): Self-esteem and guilt-and-blame
  IFQ was utilized to assess responses regarding self-esteem and guilt-and-blame. In the study, self-esteem, defined as a disposition for judgment of oneself, reflects an individual’s overall evaluation, positive or negative, of his or her own capacity, worth, and charm under the presence of obstacle to fertility. Guilt-and-blame was defined as a negative emotional experience upon feelings of violating moral standards and responsibility and of the act of censuring and making adverse statements involving gender attributes, inability, doubt, and punishment due to infertility. Self-esteem and guilt-and-blame subscales consisted of three and six items administered in the survey, respectively. Each of the items was scored from 1 (strongly disagree) to 5 (strongly agree).
4. Inventory of Socially Supportive Behaviors (ISSB; Barrera, Sandler [33]): Social support
  ISSB sought to measure social support, which was represented by the perception of emotional, informational, and tangible supportive resources received from social network including listening, caring, comforting, accompanying, providing information or opinion for infertility treatment, and financial assistance in need. Social support comprised six items and each item ranged from 1 (strongly disagree) to 5 (strongly agree).
5. Perceived Stress Scale (PSS-10; Cohen [34]): Perceived stress level
  PSS-10 aimed to measure perceived stress level that was defined as the degree to which a person perceived that demands exceed one’s ability to cope in the psychological state pertaining to temper, controllability, solvability, and tenseness. Perceived stress comprised six items, with each item ranging in a level of 1 (strongly disagree) to 5 (strongly agree).
6. Hospital Anxiety and Depression Scale (HADS; Zigmond and Snaith [35]): Anxiety and depression
  HADS was an effective tool designed for clinically predicting two dimensions of mental illness including anxiety and depression symptoms. Each single item was scored from 0 (not at all) to 3 (very often), in conformity with the original design. A summed score range of 8 to10 in this standardized measure indicated "borderline abnormal"; scores > = 11 were regarded as "abnormal"; otherwise, "normal" (0 to 7). Levels of anxiety and depression apply to the same measure standard, as mentioned.

This study applied a two-stage questionnaire administration to establish psychometric properties such as validity and reliability of the instrument. The initial questionnaire comprised 58 items measuring the aforementioned 10 constructs. First, a pilot study with a sample of 30 infertile patients was conducted. The preliminary analysis showed that the adapted questionnaire items reached favorable convergent construct validity and internal consistency reliability. Second, formal administration of the survey was performed afterward. Questionnaire items were subject to be removed from subsequent analysis because proper item deletion led to improved psychometric properties in terms of Cronbach’s alpha and factor analysis.

Composite reliability of all the constructs was higher than 0.8, with an exception of 0.64 for social stressor. Cronbach’s alpha coefficients for all the constructs were also satisfactory (> 0.7, except 0.63 for social stressor). Principal axis factor analysis with Varimax rotation extracted 10 corresponding components, accounting for 64.56% of the variance in the scores of the questionnaires (all factor loading > 0.55). Squared multiple correlation (SMC) confirmed strong correlations between observed variables and corresponding latent variables in the measurement model. Furthermore, the values of Average Variance Extracted (AVE) demonstrated that all the latent variables had favorable convergent validity (> 0.5) and discriminant validity based on the results of a Confirmatory factor analysis (CFA) [36]. All the psychometric properties demonstrated the questionnaire items constituted an effective instrument for assessing the psychological features among infertile individuals.

### Variables

Items measuring all latent variables except anxiety and depression were scored on a 5-point Likert scale of 1 to 5. Anxiety and depression were measured on a 4-point Likert scale of 0 to 3, with seven items for anxiety and depression respectively. All factors of stress were treated as exogenous variables, whereas stress level, self-esteem, and mental symptoms were considered endogenous variables. Different from other four exogenous variables, social support and treatment cognition served as the hypothetical protectors of mental symptoms. Variables regarding personal characteristics were defined in a categorical or ordinal level and served as additional information in the survey. 10 questionnaire items in social stressor, marital stressor, importance of children, self-esteem, and stress level were removed from the initial questionnaire to enhance the quality of measurement according to Cronbach’s α values and factor loadings of each item during the validity and reliability assessments. The final numbers of items, Cronbach’s alpha, and factor loadings of the total 10 latent variables are presented in Table 1.

### Statistical analysis

The main analysis method SEM was conducted using AMOS 17. SEM, a combination of statistical techniques including factor analysis, regression and path analysis, was employed because of its distinct capacity in estimating error variances from complicated measurement components and their structures, ideal for theory testing and development as a whole. SEM was used to test the measurement and structural models of all hypothesized relations among constructs delineated in Fig 2. The causal relationships among the constructs would be determined after the hypothesis testing by using SEM. Evaluation for goodness-of-fit of the hypothesized model involved examining the following criteria: absolute fit measurement, incremental fit measurement, and parsimonious fit measurement [36–39].

Conforming to the Model Generating (MG) paradigm proposed by Joreskog [40], the present research specified a tentative theory-driven model (Fig 2) and, after testing, might modify the model to achieve acceptable goodness-of-fit indexes using the same data. Overall, 48 observed variables (questionnaire items) which were extracted to 10 latent variables entered the analysis using SEM.

## Results

The descriptive analyses for all the 10 latent variables used in the model are shown in Table 1. Among all the eight constructs measured on a 5-point Likert scale, self-esteem reached the highest average score at 4.044, whereas noteworthily social support received a relatively low average of 1.780. Regarding the two mental symptoms, patients with infertility reported anxiety symptoms at a higher sum score of 6.148 compared with that of depression symptoms at 4.525. According to the norms of HADS proposed by Zigmond and Snaith [35], approximately three tenths of the infertile patients (30.8%) were categorized as borderline abnormal or abnormal level of anxiety (8–10 or > = 11, respectively). Nearly one fifth of the infertile patients (20.8%) presented borderline abnormal or abnormal level of depression (8–10 or > = 11, respectively). Similarly, abnormal level of anxiety reported by the participants was 12.8%, higher than the percentage of abnormal level of depression (8.5%). 16.5% of the infertile patients showed both borderline abnormal symptoms of anxiety and depression. 5.8% patients with infertility reached the abnormal level of both anxiety and depression.

To examine if the proposed theoretical structural model fit the data, the Modification Index (MI) of the goodness-of-fit test was used to revise the relational paths among the constructs. When MI is higher than 5, it is necessary to correct for the residual values [37–40]. Nevertheless, the present study used a stricter criterion of MI higher than 10 in modifying the model, cautiously ascertaining the revision was more literature-based than data-driven.

**Fig 3:**
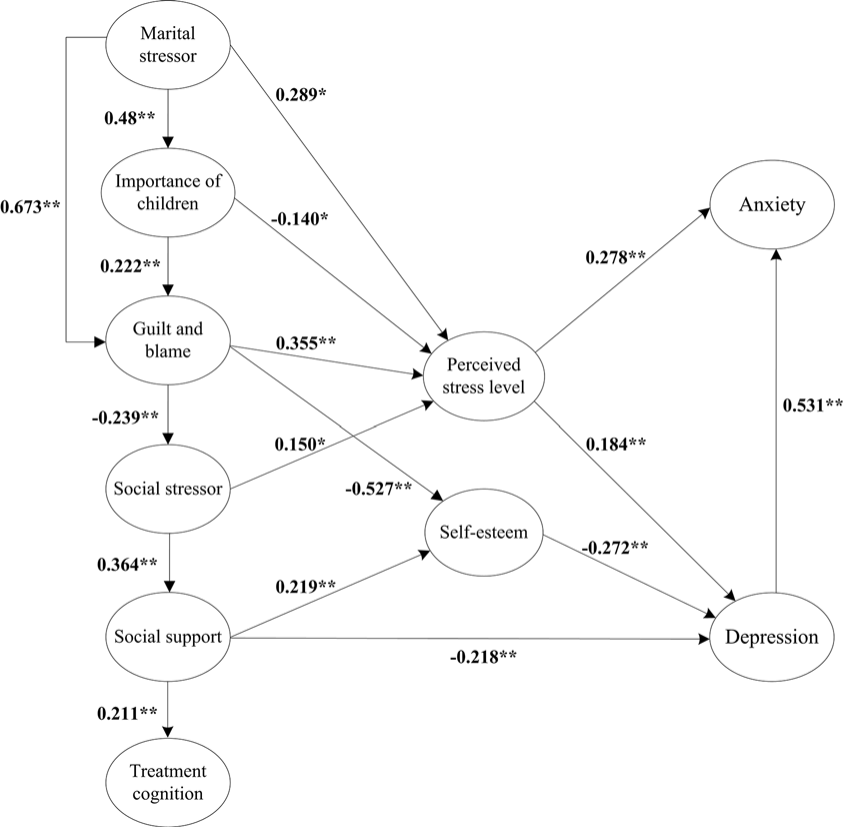
Final Model: The Stressors Stress Symptoms (SSS) Model for Patients with Infertility (*: *P*-value < 0.05, **: *P*-value < 0.001).

All the three indexes of parsimonious fit measurement showed an adequate model fit, including essential χ^2^/DF = 1.871 (<2), PGFI = 0.746 (>0.50), and PNFI = 0.764 (>0.50). Five additional indexes from incremental fit measurement and absolute fit measurement confirmed the modified model satisfactory, including IFI = 0.918 (>0.90), CFI = 0.917 (>0.90), SRMR = 0.049 (<0.05), GFI = 0.863 (>0.90; nearly passed, acceptable), and RMSEA = 0.047 (< 0.05). In short, the final model fit the data well (Fig 3).

According to the final model shown in Fig 3, factors that had a direct effect on perceived stress level included marital stressor, importance of children, guilt-and-blame, and social stressor. Factors directly affecting depressive symptoms included perceived stress level, self-esteem, and social support. Perceived stress level and depression were also two factors that contributed to anxiety symptoms. Table 2 shows direct effects, indirect effects, and total effects among all the constructs. In the ranking of direct effect size, guilt-and-blame (0.355), marital stressor (0.289), and social stressor (0.150) were the three major factors affecting perceived stress level; self-esteem (-0.272), social support (-0.218), and perceived stress level (0.184) were the three influencing factors of depression in order; depression (0.531) and perceived stress level (0.278) were the two factors positively and directly affecting anxiety in order. Nine of the eleven hypothetical paths from the initial model (Fig 2) had been confirmed. Several newly established paths not in the initial model are displayed in the final model in Fig 3, for example, the mediation effect of self-esteem between guilt-and-blame and depression.

**Table 2:** Direct and Indirect Effects among all the Constructs in the Model Tested.

	Direct Effect	Indirect Effect	Total Effect
***Treatment cognition***			
Social stressor → Treatment cognition		0.077	0.077
Marital stressor → Treatment cognition		-0.014	-0.014
Importance of children → Treatment cognition		-0.004	-0.004
Guilt-and-blame → Treatment cognition		-0.018	-0.018
Social support → Treatment cognition	0.211		0.211
***Social stressor***			
Marital stressor → Social stressor		-0.186	-0.186
Impo rtance of children → Social stressor		-0.053	-0.053
Guilt-and-blame → Social stressor	-0.239		-0.239
***Importance of children***			
Marital stressor → Importance of children	0.480		0.480
***Self-esteem***			
Social stressor → Self-esteem		0.080	0.080
Marital stressor → Self-esteem		-0.426	-0.426
Importance of children → Self-esteem		-0.121	-0.121
Guilt-and-blame → Self-esteem	-0.527	-0.019	-0.546
Social support → Self-esteem	0.219		0.219
***Guilt-and-blame***			
Marital stressor → Guilt-and-blame	0.673	0.106	0.779
Importance of children → Guilt-and-blame	0.222		0.222
***Social support***			
Social stressor → Social support	0.364		0.364
Marital stressor → Social support		-0.068	-0.068
Importance of children → Social support		-0.019	-0.019
Guilt-and-blame → Social support		-0.087	-0.087
***Perceived stress level***			
Social stressor → Perceived stress level	0.150		0.150
Marital stressor → Perceived stress level	0.289	0.238	0.527
Importance of children → Perceived stress level	-0.140	0.087	-0.053
Guilt-and-blame → Perceived stress level	0.355	0.036	0.391
***Anxiety***			
Social stressor → Anxiety		-0.110	-0.110
Marital stressor → Anxiety		0.267	0.267
Self-esteem → Anxiety		-0.144	-0.144
Guilt-and-blame → Anxiety		0.236	0.236
Social support → Anxiety		-0.147	-0.147
Perceived stress level → Anxiety	0.278	0.098	0.376
Depression → Anxiety	0.531		0.531
***Depression***			
Social stressor → Depression			-0.129
Marital stressor → Depression		0.227	0.227
Importance of children → Depression		0.027	0.027
Self-esteem → Depression	-0.272		-0.272
Guilt-and-blame → Depression		0.239	0.239
Social support → Depression	-0.218	-0.060	-0.278
Perceived stress level → Depression	0.184		0.184

## Discussions

Initiating from the theoretical construction based on relevant literature, this study then proceeded to examine a model specific to explain the relationships among infertility-related stressors and psychological symptoms by using data from 400 infertile participants. The study population can be characterized as low social support perceived and high prevalence of borderline abnormal or abnormal symptoms of anxiety and depressive (35%), both worth of attention in terms of health interventions. The findings of higher scores in anxiety and also higher percentage of abnormal anxiety symptoms, compared with those of the depressive symptoms, are similar to those of the existing literature [25, 41]. Constructs and their relational paths to mental symptoms are identified in the final model (Fig 3). Implications of the relational paths in the model confirmed from SEM require interpretations.

The effects of marital stressor and social stressor on perceived stress level have been confirmed (H2 and H3), consistent with the existing literature [21]. Since infertility is a shared stress event in the family [42], adequate understanding of infertility problem from spouses and even properly discussing and coping the problem together with spouses may eliminate the stress level of the patients. Moreover, it has been found that the attitudes of other family members, social interactions that involved children-related conversations, and social context also create effects on perceived stress in this group [10, 21]. Guilt-and-blame is found to directly influence perceived stress level (H5); this result echoes with findings from the existing research suggesting that the feelings of guilt and self-blame are popular among infertile individuals [43], especially female [6, 44]. Traditionally, social culture expects women to procreate. Women are easily blamed for pregnancy failure and the stigma impacts on perceived stress [45]. The psychological disturbances induced by guilty feelings of infertility emerge as a strong stressor [32] and may subsequently lead to depression and anxiety [43]. Self-esteem mediates the effect of social support on depression (H7 and H11). Patients diagnosed with infertility may undergo psychosocial turmoil, possibly leading to low-esteem [46, 47]. Depression as a complicated mood state characterized by helplessness and low esteem [48] could be directly explained by self-esteem. A positive relationship between social support and self-esteem is also confirmed (H7). Self-esteem increases linearly with the increment of social support. Notably, high levels of social support and self-esteem may contribute to the alleviation of the degree of mental symptoms.

Perceived level of infertility-related stress produces a direct positive effect on both anxiety (H8) and depression (H9), which parallels with findings of prior related studies [49–51]. The disease course of infertility frequently accompanies anxiety and depression. This phenomenon could be partially explained by that the uncertainty and fear of treatment failure results in anxiety and that inability to conceive leads to depression [25]. Stress that consists of personal, marital, and social stressors gives rise to anxiety and depression symptoms [10, 21, 22, 25, 52, 53]. In other words, evidence proves that perceived stress level mediates the relationships between stressors and psychological distress among this population. In addition, depression generates a markedly direct positive effect on anxiety (H10; 0.531, the highest among all the effects). Health care providers and health educators might deliberate upon incorporating psychological interventions in the meaning and impact of the sociocultural values of parenthood and children rearing to reshape personal infertility experiences [10] in reducing the risk of psychological distress occurrence. Since mental status and IVF/ET treatment outcome are reciprocally related [50], anxiety and depression require further attention in reproductive health care.

Notably, this study renders several new findings worthy of discussion. In addition to the positive effect of guilt-and-blame on stress level, the sense of guilt-and-blame also plays a key role in self-esteem (new path, Fig 3). One possible explanation is that infertile individuals, especial females, potentially treat this disease as their inescapable burden, apt to low self-esteem by guilt and self-blame [44] after receiving the diagnosis. In addition, depression is mediated by self-esteem for the effects of both guilt-and-blame and social support [23], signaling a pivotal role of self-esteem in predicting occurrence of depression among infertile patients. The more social support that infertile individuals perceive, the less likely they report depressive symptoms (new path, Fig 3). This link is concordant with finding from a recent research [43]. Traditional Confucianism still dominates the culture of highly industrialized Taiwan. "To pass on the ancestral genes" is regarded a basic yet crucial premise of filial piety in the Chinese family ethics. Thus, burden created by the failure to "sustain the ancestral line" substantially imposes unavoidable societal stress on infertile Taiwanese [54]. Hence, enhancing personal value and social support through health interventions involving psychological counseling and social work with a focus of re-constructing positive self-concept under infertility is critical to mental symptoms prevention. Lastly, social support is found to produce both direct and indirect effects on depression, yielding a room for future investigations. Recent studies have indicated the positive effects of social support on posttraumatic growth [55] and quality of life [56] among infertile women. Overall, this study suggests that taking into account various factors of stress among infertile patients may enhance the chance of identifying people at greater risk of psychological distress.

The two hypothesized paths that failed to pass the test (H1: treatment cognition-stress, H6: social support-stress, Fig 2) deserve further explanation. The average score of treatment cognition is nearly 4, indicating the acquisition of sufficient treatment knowledge among this population from various information source channels including physician and very possibly the Internet especially in this era of ubiquitous information. According to the current finding, treatment cognition, the only non-significant factor, is not an influencing factor of stress. Social support instead positively affects treatment cognition (new path, Fig 3). It is possible that infertile individuals receive more treatment knowledge through their social network, a value-adding channeling of problem coping. The ABC-X Model highlights the pivotal role of social support as resource in stress coping. Moreover, recent research has identified the direct and indirect effects of social support on infertility-related stress [6, 57, 58]. However, this study did not confirm the relationship between social support and stress perceived, but discovered a negative relationship between social support and depression as well as anxiety, mirroring the finding reported by Slade et al [41]. Different from marital and social stressors, social support is viewed as a protective factor of depression in accordance with the findings.

Certain limitations to the present study need to be addressed. First, although the study sample was selected from various teaching hospitals, extrapolation of the research findings to all other patients including ones from physician offices requires careful consideration. Second, initially Pearson *r* indicated a positive relationship between importance of children and perceived stress. However, the negative relationship between the two constructs obtained by SEM somehow could not be fully explained. Even though other constructs exhibited high effects on perceived stress, this negative relationship justifies further research on the related domain. Third, the generalizability of the findings to present year requires consideration since the characteristics of the study population may slightly change. Finally, all survey scores were based on respondents’ self-reports, thus biasing the findings conservatively.

## Conclusions

With a systematic investigation upon the relationships among stressors, perceived stress, and mental symptoms for infertile patients, the current study contributes to the knowledge body mainly by demonstrating the direct and indirect effects of marital stressor, importance of children, guilt-and-blame, social stressor, social support, perceived stress level, and self-esteem on anxiety and depression. The proposed model passed the goodness-of-fit test by using SEM. The confirmed model was named the Stressors Stress Symptoms (SSS) Model, which comprehensively delineates the factors of stress and their effects on mental symptoms including depression and anxiety. Perceived stress level and self-esteem are the two major mediators for stressors and mental symptoms. High social support and self-esteem may contribute to lessen mental symptoms. Guilt-and-blame as the strongest stressor requires further attention. The SSS model carries theoretical and clinical implications, including identifying each single yet significant stressor and predicting subsequent mental symptoms, in caring for the psychological well-beings of infertile patients.

The findings may serve as a reference for a prospective direction of advancing the infertility health care, including providing an effective instrument for screening personal, marital, and social stressors to further prevent the occurrence of severe anxiety and depression, preferably under an infertility-specific financial scheme of capitation reimbursement. Policymakers should tackle the mental illness issues by designing a holistic medicine approached and patient-centered healthcare framework, which involves psychological assessment and counseling in culturally sensitive manners, social work, and other team working for this vulnerable yet important group.
