# Correction: Investigating the Relationships among Stressors, Stress Level, and Mental Symptoms for Infertile Patients: A Structural Equation Modeling Approach

**DOI:** 10.1371/journal.pone.0144566

**Published:** 2015-12-03

**Authors:** Jong-Yi Wang, Yi-Shan Li, Jen-De Chen, Wen-Miin Liang, Tung-Chuan Yang, Young-Chang Lee, Chia-Woei Wang

The affiliation for the seventh author is not indicated. Chia-Woei Wang is also affiliated with: Graduate Institute of Clinical Medicine, College of Medicine, Taipei Medical University, Taipei, Taiwan
